# Chemical Profile and Biological Activity of *Casimiroa Edulis* Non-Edible Fruit`s Parts

**DOI:** 10.15171/apb.2017.079

**Published:** 2017-12-31

**Authors:** Wafaa Mostafa Elkady, Eman Ahmed Ibrahim, Mariam Hussein Gonaid, Farouk Kamel El Baz

**Affiliations:** ^1^Pharmacognosy Department, Faculty of Pharmaceutical Sciences & Pharmaceutical Industries, Future University in Egypt, New Cairo, Egypt.; ^2^Plant Biochemistry Department, National Research Centre, Dokki, Cairo, Egypt.

**Keywords:** Casimiroa edulis, Fatty acids, Phenolic contents, Antioxidant, Anti-inflammatory, Caco-2

## Abstract

***Purpose:*** the non-edible fruit parts of Casimiroa edulis Llave et were evaluated for their active constituents and their potential as antioxidants, anti-inflammatory and antitumor activity.

***Methods:*** Fruits peel (FP) and seeds kernel (SK) of Casimiroa edulis Llave et Lex. were extracted successively with hexane and then methanol. Fatty acids were prepared from hexane extracts and identified by GC. Total flavonoid, phenolic acids and tannins contents in methanol extracts were determined by UV spectrophotometer and identified by HPLC. Antioxidant, in-vitro anti-inflammatory activity and antitumor effect against Caco-2 cell line were determined.

***Results:*** GC analysis of hexane extracts showed that oleic acid (47.00%) was the major unsaturated fatty acids in both extracts while lignoceric acid (15.49%) is the most abundant saturated fatty acid in (FP). Total phenolic, flavonoid and tannin contents in (FP) & (SK) methanol extracts were; 37.5±1.5, 10.79±0.66 and 22.28±0.23 for (FP); 53.5±1.5mg/g, 14.44±0.32 mg/g; and 53.73±3.58 mg/g for (SK) respectively. HPLC analysis of methanol extract revealed that; the major phenolic compound was pyrogallol in (FP) and p-hydroxybenzoic acid in (SK), the major flavonoid was luteolin 6-arabinose-8-glucose in (FP) and acacetin in (SK).

***Conclusion:*** This study showed that non-edible parts of C. edulis fruit is a rich source of different phenolic compounds and fatty acids which has great antioxidant, anti-inflammatory and antitumor activities; that could be used as a natural source in pharmaceutical industry.

## Introduction

Family Rutaceae; is a small family made up of cultivated fruiting trees and medicinal herbs frequently called citrus family, it has a great economic importance because of its several edible Citrus fruits as orange, lemon, etc. Family Rutaceae is dispersed all over the world, particularly in warm climate and tropical areas, mostly found in Africa and Australia.^1^

*Casimiroa edulis* Llaveet Lex. is a non-citrus fruit belongs to this family, it is commonly known as Zapote blanco or white sapota and mainly cultivated in Mexico and Central America.* C. edulis* is widely consumed in different parts of the world for its valuable fruit;^2^ as it is a rich source of sugar, protein, ascorbic acid, phenols, carotenoids, polyunsaturated fatty acids and minerals like Fe, Cu, Zn, Ca and K.^3^ It is traditionally used in many countries as a sleep inducer as it has interesting sedative-like effects.^2^* C. edulis* leaves and seeds were found to affect blood pressure, cardiac activity aortic muscular tone,^4^ and to possess anticonvulsant activity.^5^ Methanol extract *of C. edulis* leaves also showed strong antioxidant activity.^3^

Different classes of compounds were previously separated from different parts of *C. edulis*; furocoumarins and polymethoxyflavones were isolated from the leaves that exhibited adipogenesis activity.^6^ Moreover the leaves essential oil had promising antimicrobial activity and mainly contain sesquiterpene hydrocarbons as major constituents.^7^ Zapotin; a flavanoidal compound which considered as chemo-preventive agent was isolated from the seeds; it was also chemically synthesized because of its great anticancer activity.^8^ Different compounds were also isolated from the seeds methanolic extract and showed great cardiovascular activity. ^9^

Several studies reported that; *C. edulis* can be considered as valuable plant, so the aim of this study is to evaluate the importance of the non-edible parts of *C. edulis* fruit to evaluate its chemical composition as well as antioxidant, anti-inflammatory and antitumor potential.

## Materials and Methods

### Plant material

The fruit of *C. edulis* was collected from a public garden in Helwan, Cairo, Egypt and identified by taxonomist Therese Labib, consultant in the central gardening administration, Orman garden, Giza, Egypt. Fruits were peeled (FP), seeds were separated from the fruit and the kernel was obtained after removing the seed testa (SK). Both were separately dried at room temperature. A voucher specimen (PHG-8) has been deposited in the Pharmacognosy Department, Faculty of Pharmacy, Future University in Egypt (FUE), New Cairo, Egypt.

#### Preparation of plant extract

100 gm fruit peel (FP) and 100 gm seed kernel (SK) of *C. edulis* were separately coarsely powdered and extracted with n-hexane then by methanol for 72 h using a Soxhlet extractor at 60°C. All the extracts were dried separately under reduced pressure.

#### Chemical composition 

#### GC analysis of the Fatty Acids composition of hexane extract

Hexane extracts of (FP)He and (SK)He were subjected separately to direct methylation in 1.5% sulfuric acid –methanol at 95°C for 2 h.^10^

#### Total Flavonoid, Phenolic acids &Tannins content in methanol extract of C. edulis

This was determined for the methanol extracts of (FP)Me and (SK)Me according to methods described previously.^11,12^

#### HPLC Analysis of the methanol extracts

The phenolic and flavonoid compounds of (FP)Me and (SK)Me of* C. edulis* were extracted according to the method described by Mattila *et al.*^13^

#### Biological activity for methanol and hexane extracts

#### Antioxidant activity of C. edulis extracts using ABTS, DPPH and Total antioxidant activity 

It was carried out according to Arnao* et al*.,^14^ Ye *et al.* method.^15,16^

#### In vitro Antitumor activity 

The activity was tested on Caco-2 cell line using sulforhodamin B assay.^17^

#### In vitro Anti-inflammatory activity using bovine albumin serum

This was tested using the method of Rahman *et al*.^18^

#### Statistical analysis

All result is expressed as mean value of three replicate. Data were statistically analyzed through analysis of variance (ANOVA) and Duncans test at P>0.01 using CoStat Statistics Software.

## Results and Discussion

### Chemical composition 

#### Fatty acids composition of C. edulis hexane extracts

"Table 1" showed that Both (FP)He and (SK)He extracts revealed high percentage of total unsaturated fatty acids 71.15% and 94.20% respectively. The monounsaturated fatty acids oleic acid (omega-9) is the most abundant in both extracts; (36%) in (FP)He and (47%) in (SK)He; Also palmitoleic acid was found in (FP)He (20%) and (SK)He (21%). Furthermore, the hexane extracts showed the presence of different long chain mono and poly unsaturated fatty acids. The unsaturated fatty acids have a great role in decreasing the risk of certain cancers, as colon cancers, breast and prostate.^19^

(FP)He has higher percent of total saturated fatty acid 28.85% than that in (SK)He 5.8%; lignoceric acid 15.49% was the major in (FP)He while palmitic acid 3.01% was the highest in (SK)He; these fatty acids play important role in increasing LDL cholesterol level.^20^

**Table 1:** GC analysis of unsaturated fatty acid% in hexane extracts

**Unsaturated fatty acid**		**Fatty acid %**	
		**(FP)He extract**	**(SK)He extract**
C_14:1_	Myristoleic	1.64	0.5
C_15:1_	Pentadecanoic acid	1.29	0.32
C_16:1_	Palmitoleic acid	20.00	21.00
C_17:1_	Heptadecanoic acid	0.35	0.63
C_18:1_	Oleic acid	36.00	47.00
C_18:1_	Vaccenic acid	0.3	ND
C_18:2_	Linoleic acid	2.18	9.00
C_18:3_	α-Linolenic acid	2.56	1.09
C_18:3_	γ-Linolenic acid	ND	9.01
C_20:2_	Eicosadienoic acid	1.09	2.20
C_20:3_	Eicosatrienoic acid	1.28	1.9
C_22:1_	Erucic acid	1.89	ND
C_24:1_	Nervonic acid	2.57	1.55
**Saturated fatty acid**		**(FP)He extract**	**(SK)He extract**
C_6:0_	Caproic acid	0.77	ND
C_8:0_	Caprylic acid	0.60	ND
C_10:0_	Capric acid	0.13	ND
C_11:0_	Undecylic acid	0.2	ND
C_12:0_	Lauric acid	0.67	0.15
C_13:0_	Tridecylic acid	2.47	ND
C_14:0_	Myristic acid	3.4	0.2
C_15:0_	Pentadecylic acid	2.06	0.63
C_16:0_	Palmitic acid	1.23	3.01
C_17:0_	Heptadecanoic acid	ND	1.30
C_21:0_	Heneicosylic acid	0.45	ND
C_22:0_	Behenic acid	1.28	0.3
C_23:0_	Tricosylic acid	0.1	0.21
C_24:0_	Lignoceric acid	15.49	ND
Total mono-unsaturated fatty acid%		64.04	71
Total poly-unsaturated fatty acid %		7.11	23.2
Total saturated fatty acid %		28.85	5.8

ND: not detectable (FP)He: fruit peel hexane extract (SK)He: seed kernel hexane extract

Lipid profile presented in "Table 1" showed that both (FP)He and (SK)He extracts have great percentage of unsaturated fatty acids more than the saturated one; this indicate that the non-edible parts of *C. edulis* can be considered as a valuable natural source that offer a way of increasing the availability of unsaturated fatty acids especially oleic, palmiotleic, linoleic and γ-linolenic acid. Previous studies proved that those acids have a role in inflammation suppression.^20^

#### Total Flavonoid, Phenolic acids & Tannins contents in C. edulis methanol extract 

The results of qualitative analysis of both extracts (FP)Me & (SK)Me revealed the presence of considerable amount of secondary metabolites which could be an indication for their pharmaceutical potential. The results in "Table 2" showed that they are more abundant in (SK)Me than that in (FP)Me.

#### HPLC Analysis of phenolic compounds and flavonoid contents in C. edulis methanol extracts

"Table 3" recorded that the (FP)Me and (SK)Me extracts contained different phenolic and flavonoid compounds.

**Table 2:** Total phenolic acid, flavonoid and tannin contents in *C. edulis* methanol extracts

**Methanol extract**	**Phenolic (mg/g) DW**	**Flavonoids (mg/g) DW**	**Tannins (mg/g) DW**
(FP)Me	37.5±1.5^b^	10.79^±^0.66^b^	22.28±0.23^b^
(SK)Me	53.5±1.5^a^	14.44±0.32^a^	53.73±3. 58^a^
LSD	1.9	5.6	9.5

DW: dry weight, (FP)Me: fruit peel methanol extract, (SK)Me: seed kernel hexane extract

**Table 3:** HPLC analysis of the phenolic and flavonoids compounds in *C. edulis* methanol extracts

**Phenolic compounds**	**(FP)Me (mg/100g) DW**	**(SK)Me (mg/100g) DW**
3,4,5-methoxycinnamic acid	3.43	37.34
4-amino benzoic acid	79.86	4.86
Benzoic acid	252.60	251.11
Caffeic acid	15.38	48.76
Catechein	169.77	240.81
Catechol	230.60	190.87
Chlorogenic acid	175.36	410.98
Cinnamic acid	6.36	24.44
Ellagic acid	52.37	133.42
Epicatechein	176.30	60.97
e-vanillic acid	457.57	344.81
Ferulic acid	53.20	58.32
Gallic acid	21.56	28.94
Iso-ferulic acid	100.11	22.34
*p*-coumaric acid	52.04	55.63
*P*-hydroxy benzoic acid	185.72	1571.13
Protocatechuic acid	79.86	89.72
Pyrogallol	1846.16	695.98
Reversetrol	7.00	14.45
Rosmarinic acid	30.37	11.27
Salycilic acid	18.39	60.80
Vanillic acid	53.48	49.70
α- coumaric acid	7.75	36.95
**Flavonoids compounds**	**(FP)Me (mg/100g) DW**	**(SK)Me (mg/100g) DW**
Luteolin-6-arabinose-8-glucose	1907.92	1242.72
Luteolin-6-glucose-8-arabinose	537.94	561.91
Apigenin-6-arabinose-8-galactose	97.63	41.01
Apigenin-6-rhamnose-8-glucose	322.24	592.74
Apigenin-6-glucose-8-rhamnose	823.66	129.61
Apigenin-7-*O*-neohespiroside	-	17.94
Apigenin-7-*O*-glucose	-	54.66
Luteolin-7-*O*-glucose	-	26.09
Kampferol-3,7-dirhamoside	-	47.02
Luteolin	1103.24	150.63
Acacetin	103.93	2560.78
Naringin	3.48	291.92
Rutin	238.18	181.26
Hespirdin	196.86	-
Quercetrin	25.10	37.75
Quercetin	35.92	298.65
Kampferol	6.66	14.70
Hespirtin	10.45	26.26
Apigenin	0.48	87.31
Rhamnetin	2.58	66.26
**Total identified compounds**	16	19

Twenty three phenolic compounds were identified in both (FP)Me and (SK)Me by comparison with authentic reference compounds. In (FP)Me pyrogallol is the most abundant phenolic compound 1846.16 mg/100g followed by e-vanillic acid 457.57 mg/100g, benzoic acid 252.6 mg/100g and catechol tannins 230.6 mg/100g. The major phenolic compound in (SK)Me was *P*-hydroxy benzoic acid 1571.13 mg/100g followed by pyrogallol 695.98 mg/100g then cholinergic acid 410.98 mg/100g and e-vanillic acid 344.81 mg/100g.

The total flavonoid compounds identified in (FP)Me extract was 16 compounds the major compound was luteolin 6-arabinose-8-glucose 1907.92 mg/100g.

#### Biological activity

#### Antioxidant activity of C. edulis extracts

**Figure 1:**
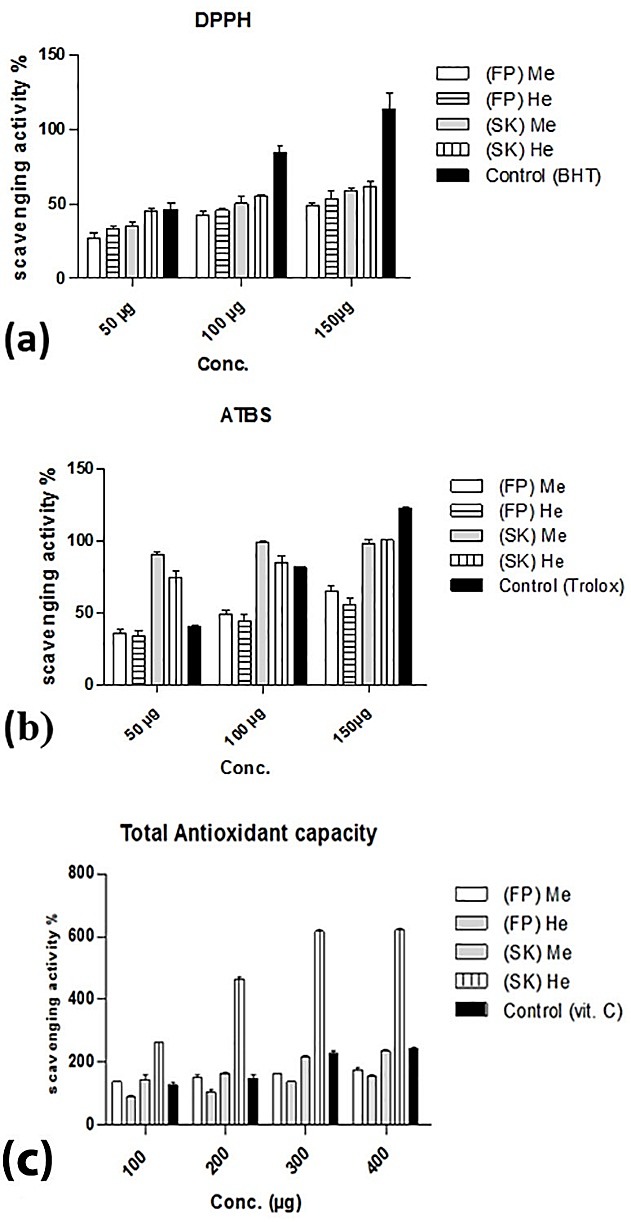
Antioxidant activity of *C. edulis* extracts(FP) Me: fruit peel methanol extract, (SK) Me: seed kernel hexane extract, (FP) He: fruit peel hexane extract, (SK) He: seed kernel hexane extract

The antioxidant activity of the methanol and hexane extracts of both (FP) and (SK) was evaluated using the ABTS and DPPH free radical-scavenging assay; “Figure 1a and 1b” showed that the (SK) extracts has higher antioxidant activity than the (FP) extracts, this may be attributed to the higher unsaturated fatty acid, phenolic & flavonoid contents.

On the other hand the results showed that most powerful antioxidant activity is presented in the (SK)He extract "Figure 1c"; this could be due to the high percentage of the unsaturated fatty acids 94.2% "Table 1" especially oleic acid which has great role in protection of cell membranes from free radicals.^21^

The antioxidant activity was also previously reported in the edible parts and leaves methanol extract of *C. edulis*.^3^

#### In vitro Anti-inflammatory activity 

**Figure 2:**
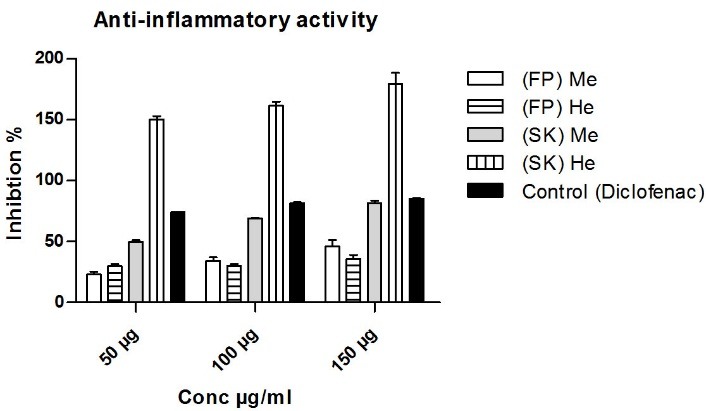
Anti-inflammatory activity of *C. edulis* extracts
(FP) Me: fruit peel methanol extract, (SK) Me: seed kernel hexane extract, (FP) He: fruit peel hexane extract ,(SK) He: seed kernel hexane extract

Results in "Figure 2" showed that the (SK)He extract at different doses (50, 100 and 150µg/ml) has the most potent anti-inflammatory activity compared with (Diclofenac) as control drug. This effect may be due to the high percentage of the unsaturated fatty acids in (SK)He extract;^22^ the potential anti-inflammatory activity of the methanol extracts can be also attributed to the presence of higher percentage of phenolic contents.

#### Antitumor activity of C. edulis extracts 

**Figure 3:**
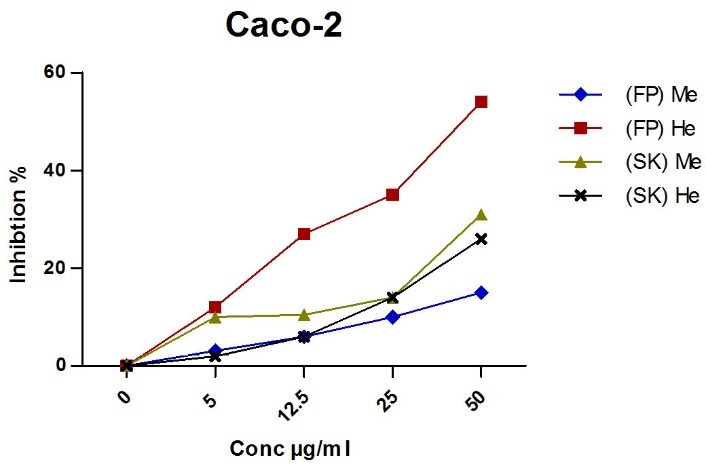
Antitumor activity of *C. edulis* extracts
(FP) Me: fruit peel methanol extract, (SK) Me: seed kernel hexane extract, (FP) He: fruit peel hexane extract, (SK) He: seed kernel hexane extract

"Figure 3" reveled that (FP) and (SK) extracts have certain inhibition effect against the Caco-2 cell line but the most active extract is the (FP)He extract when compared with reference drug doxorubicin, where the IC_50_ is 45 µg/ml.

## Conclusion

*C. edulis* non edible fruit parts could be considered as a valuable source for different useful metabolites as unsaturated fatty acid in the hexane extract and poly-phenolic, flavonoids and tannins in methanol extract; both extracts revealed great importance as antioxidant, anticancer and anti-inflammatory activities. Thus the non-edible part of fruit which is considered as waste product may be phyto-therapeutically used. However; further *in vivo* studies are required to authenticate such biological activities in order to formulate safe effective pharmaceutical herbal product.

## Ethical Issues

Methods are done after approval of the research ethics committee; the approval form has serial no. REC-FPSPI-5/34.

## Conflict of Interest

The authors declare no conflict of interests.
